# Differential gene expression in an elite hybrid rice cultivar (*Oryza sativa, L*) and its parental lines based on SAGE data

**DOI:** 10.1186/1471-2229-7-49

**Published:** 2007-09-19

**Authors:** Shuhui Song, Hongzhu Qu, Chen Chen, Songnian Hu, Jun Yu

**Affiliations:** 1 Key Laboratory of Genome Science and Information, Beijing Institute of Genomics, Chinese Academy of Sciences, Beijing 101300, China; 2 Department of Biology, Graduate University of the Chinese Academy of Sciences, Beijing 100094, China

## Abstract

**Background:**

It was proposed that differentially-expressed genes, aside from genetic variations affecting protein processing and functioning, between hybrid and its parents provide essential candidates for studying heterosis or hybrid vigor. Based our serial analysis of gene expression (SAGE) data from an elite Chinese super-hybrid rice (*LYP9*) and its parental cultivars (*93-11 *and *PA64s*) in three major tissue types (leaves, roots and panicles) at different developmental stages, we analyzed the transcriptome and looked for candidate genes related to rice heterosis.

**Results:**

By using an improved strategy of tag-to-gene mapping and two recently annotated genome assemblies (*93-11 and PA64s*), we identified 10,268 additional high-quality tags, reaching a grand total of 20,595 together with our previous result. We further detected 8.5% and 5.9% physically-mapped genes that are differentially-expressed among the triad (in at least one of the three stages) with *P*-values less than 0.05 and 0.01, respectively. These genes distributed in 12 major gene expression patterns; among them, 406 up-regulated and 469 down-regulated genes (*P *< 0.05) were observed. Functional annotations on the identified genes highlighted the conclusion that up-regulated genes (some of them are known enzymes) in hybrid are mostly related to enhancing carbon assimilation in leaves and roots. In addition, we detected a group of up-regulated genes related to male sterility and 442 down-regulated genes related to signal transduction and protein processing, which may be responsible for rice heterosis.

**Conclusion:**

We improved tag-to-gene mapping strategy by combining information from transcript sequences and rice genome annotation, and obtained a more comprehensive view on genes that related to rice heterosis. The candidates for heterosis-related genes among different genotypes provided new avenue for exploring the molecular mechanism underlying heterosis.

## Background

Heterosis is defined as advantageous quantitative and qualitative traits of offspring over their parents, and the utilization of heterosis principles has been a major practice for increasing productivity of plants and animals [1]. A considerable amount of efforts have been invested in unraveling genetic basis of heterosis in rice (*Oryza sativa, L*) and it was explained mainly by mechanisms such as dominance [2] and epistasis [3]. Although many investigators favored one hypothesis over another, biological mechanisms of rice heterosis may not be fully characterized based on genetic approaches alone, especially based on classical genetic concepts.

Recently, it has been reported that differentially-expressed genes between hybrids and their parental inbreeds are correlated with heterosis [4,5]. In wheat, a variety of differentially-expressed genes including transcription factors and genes involved in metabolism, signal transduction, disease resistance, and retrotransposons were detected responsible for heterosis by using a differential display technique [6,7]. Even ribosomal proteins have been scrutinized since they are indicators of translation activities and plastid biogenesis [8]. Various techniques have been applied to pin down genes involved in heterosis, such as a variety of sequence-based and hybridization-based methods; some have yielded interesting candidates and others proposed expression patterns of these candidates [5,9]. For instance, a hybrid-specific expressed gene AG5 (a RNA-binding protein) in wheat was identified [10]. Another study on gene generated expression profiles of an elite rice hybrid and its parents at three stages of young panicle development by using a cDNA microarray consisting of 9,198 ESTs and the result pointed to a significant mid-parent heterosis [11]. Nevertheless, it is necessary to generate more data in large-scale, taking the advantage of the fast advancing genomic technology.

SAGE technology is a sequence-based approach for investigating gene expression in large-scale and allows much deeper sampling than EST (expressed sequence tag)-based approaches. It has proven to be a very powerful method for large-scale discovery of new transcripts, acquisition of quantitative information of expressed transcripts, and the quantitative comparison between libraries [12-14]. The technique has been used extensively in animal systems including human and mouse, and more particular in cancer research where several hundred libraries and nearly 7 million SAGE tags have been obtained [13,15]. In plant, several studies have employed this methodology for transcript profiling in Arabidopsis [16,17] and rice [18,19]. However, a bottleneck of SAGE is tag-to-gene mapping, which refers to the unambiguous determination of the gene represented by a SAGE tag. Other limitations include lack of accurate genomic sequences and adequate amount SAGE data. Therefore, encouragements should be given to studies that generated publicly available data since heterosis is not simply a manifestation of a few seemingly important genes but many.

We have been studying the rice genome with a particular interest in the molecular mechanism of heterosis as part of the Super-hybrid Rice Genome Project (SRGP), focusing on an elite super-hybrid (*Liang-You-Pei-Jiu, LYP9 *[20]) and its parental lines, using gene expression technology, including EST and SAGE techniques. The objective of our current work was to recover more sequence tags (gene expression information) from our previous SAGE study [21]. In our new analysis, SAGE tags were mapped to two newly annotated genome assemblies, paternal cultivar (*93-11*) and maternal cultivar (*Pei-Ai 64s*, *PA64s*) (BGI unpublished data) [22,23]; the latter was not available when we carried out the first analysis. Prefect matches of SAGE tags to their own genome sequences allowed us to map more tags in a very significant way: twice as much tags were mapped as compared to the previous result. We also used three types of transcripts, including full-length cDNA (FL-cDNA) [24], expressed sequence tags (ESTs) [25,26], and UniGene data as well as a new strategy in the current analysis.

## Results

### The dataset

We obtained a total of 465,164 SAGE tags from nine SAGE libraries constructed in parallel from the three major rice tissues at distinct growth stages for the super-hybrid rice (*LYP9*) and its parental (*93-11 and PA64s*) cultivars. These libraries were made with mRNA isolated from (1) leaves at the milky stage of rice grain maturation, (2) panicles at the pollen-maturing stage, and (3) roots at the first tillering stage [21]. By using more stringent sequence-analysis criteria in a quality-improving protocol, we removed contaminated tags matched to cloning linkers, vectors, and simple repeats, and obtained 68,462 unique empirical tags; this number is 21 tags less than the previous dataset due to more stringent filters. Of these unique tags, 30,595 (44.7%) tags were observed more than once. The distribution of the mapped tags among different libraries is summarized in Table 1. We deposited all the original SAGE data in NCBI's Gene Expression Omnibus [27] and these data are accessible through GEO Series accession number GSE8048.

**Table 1:** Summary of mapped tags among nine libraries

Library^a^	Total Tags	Unique Tags	Mapped Tags^b^	% Mapped	Copy Number Distribution of Mapped Tags				
					
					>= 100	21–99	6–20	2–5	1
N1	69545	22887	9898	43.2	24	235	1240	3922	4477
N2	52313	15396	8102	52.6	38	197	795	2950	4122
N3	48196	18073	8299	45.9	12	154	885	3103	4145
P1	47058	11868	5531	46.6	39	158	555	1856	2923
P2	46814	13922	6352	45.6	40	176	622	2193	3321
P3	67638	19586	8392	42.8	27	257	1099	3037	3972
L1	68546	23176	10299	44.4	24	224	1178	3942	4931
L2	36209	9866	5356	54.3	40	133	552	1819	2812
L3	28845	10863	5480	50.4	6	78	468	1817	3111
Total	465164	68462	20595	-----	250	1612	7394	24639	33814

^a ^P, N, and L stand for *PA64s*, *93-11*, and *LYP9*, respectively. Numbers 1, 2, and 3 denote libraries made from materials of panicles at the pollen-maturing stage, leaves at the milking stage, and roots at the first tillering stage, respectively. ^b ^Mapped tags refer to those that mapped to the virtual transcripts based on predicted genes that are (a) supported by transcripts that have authentic 3'-UTR sequences and (b) lacking supporting evidence but defined by adding an artificial 3'-UTRs).

### Evaluation dataset, virtual tags, and mapped tags

To obtain an evaluation dataset, we constructed a PCUE (Predicted genes, FL-cDNA, UniGene, and EST) database based on available genomic resources (see Materials and Methods). We classified 41,072 predicted genes of *93-11 *into three sets: (1) 21,676 (53%) supported by one or more transcripts, i.e. by any of three pieces of supporting evidence (or types of transcripts) – FL-cDNA, UniGene, and EST, (2) 19,396 without supporting evidence, and (3) 10,702 supported by all three types of transcripts. This evaluation dataset contains 2,480 test tags from (3) and satisfies all five quality criteria (see Materials and Methods; Table 2).

**Table 2:** Dataset for evaluating tag assignment

Dataset	Subset	Total	w/o Tags^a^	w/Tags	Hits^b^	%
cDNA	cDNA	2480	0	2480	2480	100
Unigene^c^	Unigene	2806	3	2803	2627	93.62
	Uni-S	2712	1	2711	2598	95.80
	Uni-N	94	1	93	29	30.85
	UniBest	2480	0	2480	2414	97.34
	Max-Length	2480	0	2480	2411	97.22
EST^c^	EST	54764	3597	51167	36484	66.62
	EST-S	26242	1631	24611	18788	71.60
	EST-A	2749	182	2567	1665	60.57
	EST-N	21169	1592	19577	12702	60.00
	EST-B	4604	192	4412	3329	72.31
	ESTBest	2480	19	2461	1842	74.27
	Max-Length	2480	19	2461	1858	74.92
Predicted^d^	Predicted	2480	44	2436	415	16.73
	P-100	2480	26	2454	787	31.73
	P-200	2480	9	2471	1308	52.74
	P-300	2480	4	2476	1457	58.75
	P-400	2480	2	2478	1181	47.62
	P-500	2480	1	2479	869	35.04

^a ^Numbers of cDNA sequences that do not have tags due to the absence of *Nla*III sites. ^b ^Numbers of virtual tags that matched to our empirical dataset. ^c ^Capital letters stand for transcripts that have 3' polyA signal (S), 3' polyA tail (A), both the signal and the tail (B), and neither (N), respectively. ^d ^Predicated gene models and extended lengths (bp) from stop codon (P-100 to P-500).

**Figure 1:**
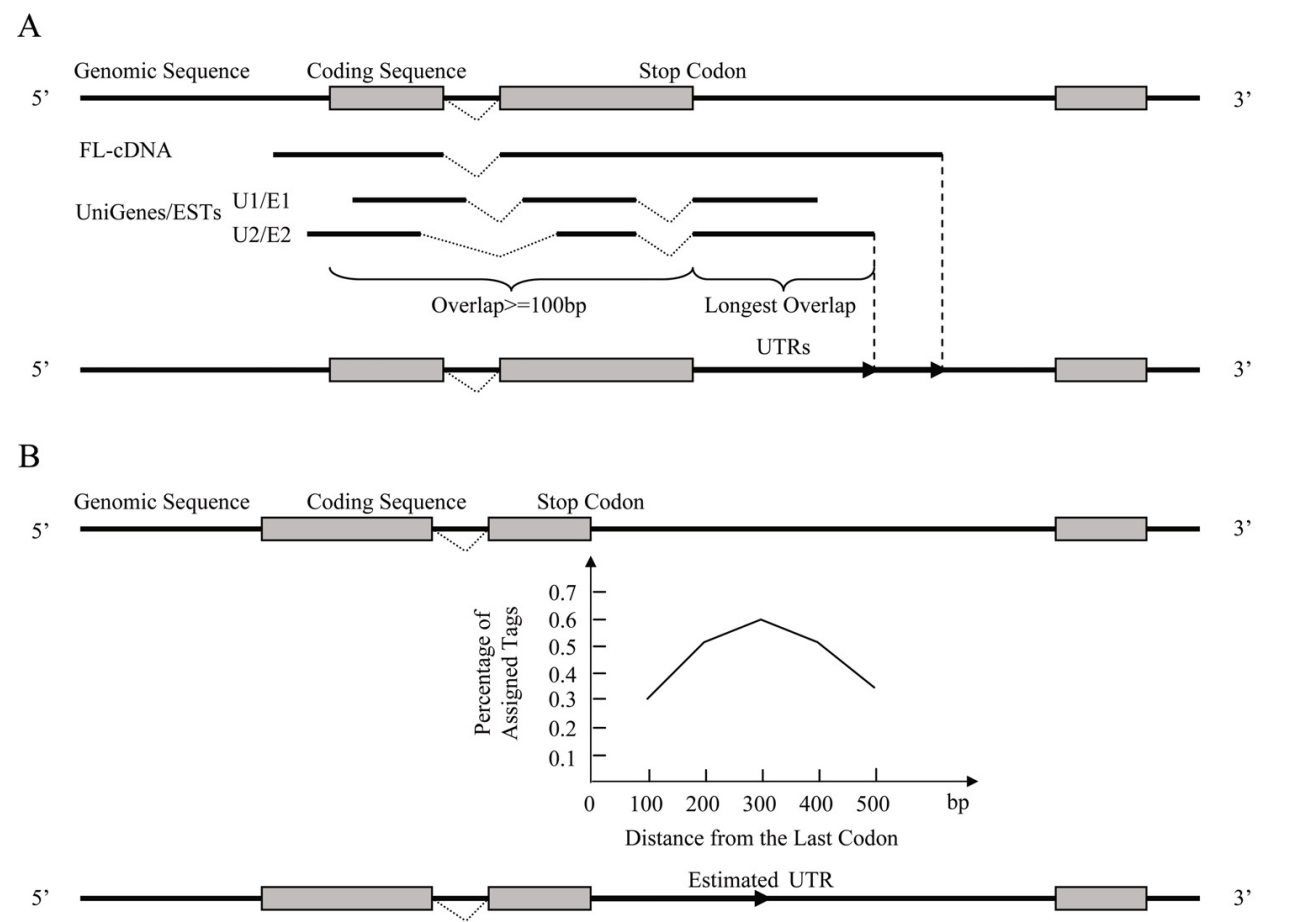
**Description of the strategy used to construct the conceptual transcript**. The high-quality genome assembly of *93-11 *(*Oryza sativa *L. subsp. *indica*; [48] and a collection of transcriptome information (FL-cDNA, UniGene, and ST; see Materials and Methods) were used for the construction of virtual transcripts. When the transcript sequences extend beyond the predicated coding sequence were available, the UTR sequences were aligned and determined (A). When the information was not available, the theoretical 3' UTR sequences were determined based on a stepwise (100-, 200-, 300-, 400-, and 500 bp) assessment of the genome sequences and added after the stop codons (B). Nearly 58.7% of the assigned tags have a 3'-UTR length of 300 bp.

**Figure 2:**
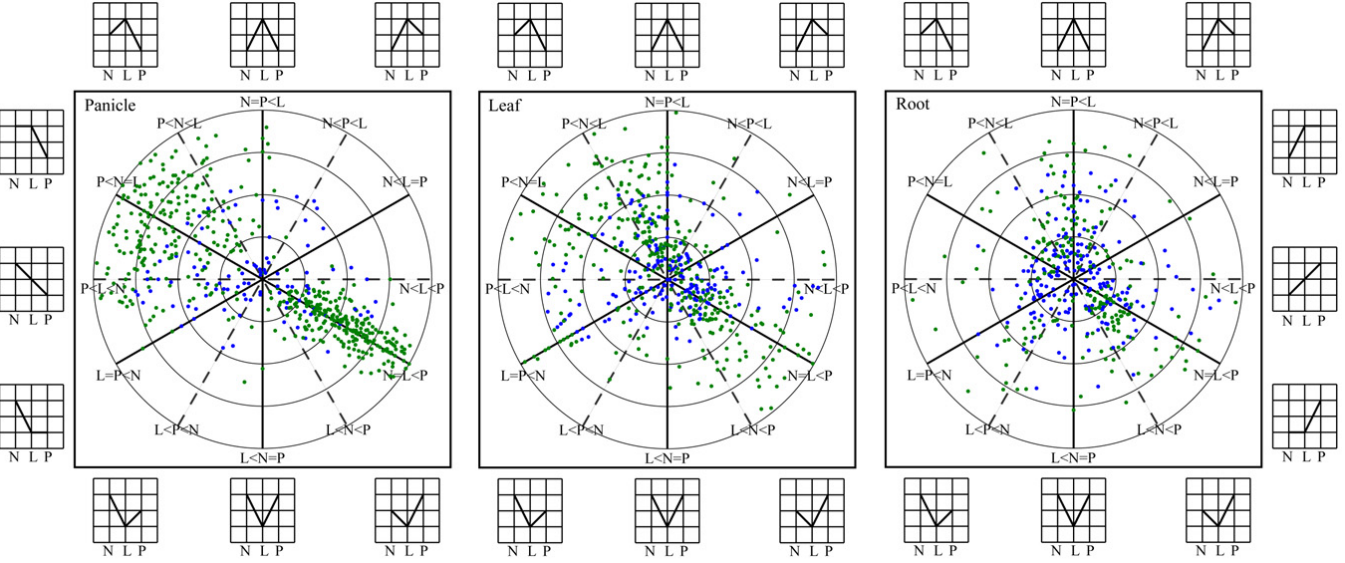
**Expression patterns and fold changes of differentially-expressed genes**. Differentially-expressed genes in panicle, leaf, and root, among *93-11 *(N), *PA64s *(P), and their F1 hybrid *LYP9 *(L) are shown. Twelve different patterns were labeled in each slice and their graphical indicators were displayed surrounding the three panels. The radius at which a gene is plotted represents log_2 _of the fold change between the high and low values among three rice cultivars, and the angle represents the relationships between *LYP9 *and its parents. Differential expressed genes with significance intervals of 0.01 <*P *< 0.05 and *P *< 0.01 are shown in blue and green, respectively. Only tags that exhibited changes of <16-fold are plotted since those beyond the fold value are very limited in numbers (listed in Additional file 5). Note (1) genes harbored by the five patterns above the horizontal lines in each panel are up-regulated (positive heterosis) in hybrid, (2) genes in the five patterns in each panel below the horizontal lines are down-regulated (negative heterosis) in hybrid, and (3) two mid-parent patterns are on the horizontal lines.

In order to define virtual tags, we need to handle two classes of virtual transcripts based on predicted genes: (1) supported by transcripts that have actual 3'-UTR sequences (Figure 1A) and (2) without supporting evidence but defined by adding an artificial 3'-UTRs (Figure 1B). From the first class, we categorized 13 different groups of virtual tags based on variable 3' UTR sequence features (in Table 2). We also found that the virtual tags constructed from the longest UniGene (Unimax, 97.22%) and the longest EST (ESTmax, 74.92%) had better yield in matching the virtual tags to the test tags, largely due to their longer 3'-UTRs. As a comparison, the virtual tags constructed from the Uni-S and EST-S groups that possessing poly (A) signals had slightly poorer but significant yields – 95.80% and 71.60%, respectively. For the second class, we need to choose a length range for artificial UTRs that are to be added to the predicted genes. For 19,079 non-redundant FL-cDNAs (see Additional file 1: UTR Size distribution), whose 3-UTRs have a distinct length distribution with a mean of 422 bp and a median of 295 bp, we decided to use a 100-bp window and an optimal length range of 300 bp. The four sets of virtual tags, including cDNA, Unimax, ESTmax, and predicted genes with 300 bp 3'-UTR, were used for further analyses (Table 2).

We assigned 20,595 unique tags to 19,961 predicted genes (Table 3) in three types: (1) 16,757 (81.36%) unambiguous tags, (2) 3,316 (16.10%) tags physically-mapped to 1,668 genes (two or more different tags assigned to the same predicted genes), and (3) 698 (3.39%) tags physically-mapped to 1,536 genes (each tag assigned to multiple genes). Among these mapped tags, 16,430 (80%) were supported by transcripts and 4,341 (20%) were not supported by known evidence; the latter are largely hypothetical transcripts that are either expressed at lower level or specific to certain tissues or developmental stages (based on microarray and EST analyses of our own data; data not shown). This process led to a more rigorous tag-to-gene assignment, allowing us to gain 10,268 additional tags, compared to our previous results. In addition, we found that 1,610 previously mapped tags were absent in the current data, and the missing tags were filtered out by the more stringent criteria used in this study that resulted in a removal of 1,649 FL-cDNAs as compared to the previous data set. There were 45,025 unmapped tags that did not satisfy our stringent criteria (see Materials and Methods for details).

**Table 3:** Mapped tags and supporting evidence

Type^a^	Mapped Tags (%)	T-supported^b^		P-supported^b^		Total Genes
			
		>1	= 1	>1	= 1	
1-1	16757(81.36%)	10087	2708	1921	2041	16757
n-1	3316(16.10%)	2476	796	26	18	1668
1-n	698(3.39%)	314	49	191	144	1536
Total	20595	12877	3553	2138	2203	19961

^a ^1-1, one tag that was mapped to a single gene; n-1, multiple tags that were mapped to a single gene; 1-n, one tag that was mapped to multiple genes. ^b ^T-supported tags are those mapped to genes with known transcripts and P-supported tag are those mapped to predicted gene models.

### Differentially-expressed genes among twelve distribution patterns

We defined differentially-expressed genes by calculating *P *values between any two libraries using a previously reported statistic method [28]; the process yielded 1,751 (8.5%) and 1,216 (5.9%) significant differentially-expressed genes with *P *values of < 0.05 and < 0.01, respectively (Table 4). In the process of summarizing overall expression profiles, regardless the origin of tissues, we found 781, 360, and 324 differentially-expressed genes from pair-wise comparisons of *LYP9 *versus *PA64s *(L *vs. *P), *LYP9 *versus *93-11 *(L *vs. *N), and *LYP9 *versus both parental cultivars (both) at a less stringent threshold (*P *< 0.05), respectively. There is an obvious bias – the genes with paternal-like expression (PLE; L *vs. *P) are twice as much as those with maternal-like expression (MLE; L *vs. *N). This bias suggests that *LYP9 *possesses more differentially-expressed genes from *PA64s *than from *93-11*, regardless whether they are up-regulated or down-regulated; in other word, *LYP9 *is more similar to *93-11 *than to *PA64s *in its overall gene expression.

**Table 4:** Differentially-expressed genes with significance ^a^

		Tag						
			
		P < 0.05			P < 0.01			Microarray-confirmed
			
	Tissue	Total	Up/Down (>= 2)^b^	Up/Down (>1)^b^	Total	Up/Down (>= 2)^b^	Up/Down (>1)^b^	Total/<0.05/<0.01^c^
N *vs *L	Panicle	371	99/80	188/167	123	33/25	52/66	1335/133/75
	Leave	411	130/64	231/126	199	81/37	124/51	
	Root	283	80/58	148/112	113	39/29	61/44	
	Panicle	666	136/238	265/332	558	123/220	221/281	
P *vs *L	Leave	476	157/84	272/179	319	131/66	194/108	1209/142/35
	Root	346	81/88	155/162	185	47/56	80/89	
	Panicle	322	91/68	175/134	91	32/16	46/42	
Both	Leave	286	121/39	194/77	125	76/21	97/29	232/53/8
	Root	194	65/36	102/73	65	31/16	37/28	
	Panicle	715	144/250	278/365	590	124/229	191/305	
Total	Leave	601	166/109	309/228	393	136/72	221/130	2312/222/102
	Root	435	96/110	201/201	233	55/69	104/105	

^a ^We listed tags that have P-value less than 0.05 and 0.01 as significant thresholds for the dataset, and divided into three categories: *PA64s vs*. *LYP9 *(P *vs. *L), *93-11 vs. LYP9 *(N *vs. *L), and the overlapped tags (Both). The statistics was based on the Audic and Claverie test statistic (IDEG6, ). ^b ^Up/Down are calculated with L/[(P+N)/2] for up-regulated tags and [(P+N)/2]/L for down-regulated tags. ^c ^The microarray data were extracted from experiments performed in our laboratory for a parallel analysis. Total consistent and significant gene numbers are listed

We further examined the profiles of differentially-expressed genes by classifying them into 12 different distribution patterns, displayed separately according to different tissues, and plotted the intensity of gene expression as fold changes (less than 16-fold) at *P *< 0.05 and *P *< 0.01 (Figure 2). There were 686, 568, and 413 genes differentially-expressed in panicles (see Additional file 2), leaves (see Additional file 3), and roots (see Additional file 4), among the triad at *P *< 0.05, respectively. The corresponding numbers were 599, 393, and 240 at *P *< 0.01. Genes that show changes of >16-fold and genes that only assigned to PA64s are also listed (see Additional file 5). In order to describe the gene distribution clearly according to their relationship between the hybrid and its parents, we partitioned the twelve distribution patterns into three basic categories: over-dominance (the top four slices), under-dominance (the bottom four slices), and mid-parent (the four slices divided by the horizontal line).

From the overall distribution of differentially-expressed genes with higher *P *values (*P *< 0.01), we made several observations among the samples. First, gene distribution pattern in panicles is rather distinct and more biased than that in the other two tissues, in such a way that most of the down-regulated genes are very paternal-like (or almost identical to *93-11*, N = L < P) and the up-regulated genes are rather dispersive (not focused along the solid line of N = L > P). The dispersiveness suggested that most of these genes are roughly paternal-like but their expression levels are approximating toward either the hybrid (*LYP9*) or the mid-parent in a quantitative manner. We speculate that this obviously restricted distribution in panicles may be either due to one or both the following possible biases. One bias may come from thermo-sensitive male sterility unique to the maternal cultivar, *PA64s*, where germline-related genes may be crippled in their overall gene expression though epigenetic mechanisms. The other possible bias may be resulted from incompatibility between alleles from the parental lines, which may cause a rather major regulatory effect for the majority of genes, such as DNA methylation in germline tissues. Second, the distribution of genes in leaves and roots are somewhat similar, especially among the down-regulated genes, and fold changes of these down-regulated genes are not as apparent as those in panicles. However, the distributions of up-regulated genes in the two tissues are rather distinct, where the up-regulated genes in leaves are biased toward over-dominant expression albeit a minority of the genes is found spreading toward mid-parent. In roots, the up-regulated genes, though they are rather smaller in number as compared to panicles and leaves (101 genes, Table 4), are mostly over-dominant. Finally, in the process of summarizing gene distributions in the twelve patterns, we found that a minority of the differentially-expressed genes (25 to 45%) exhibited additive expression (P > L > N and N > L > P; genes that were plotted on the horizontal lines), whereas the majority of the genes, 380 (55%), 408 (72%), and 309 (75%), are non-additive in panicles, leaves, and roots, respectively. Among the sum of these non-additive genes in all three tissues, 552 genes showed over-dominant expression, and a smaller amount, 394 genes, were found under-dominantly expressed. In addition, 115 and 32 genes are expressed at the same level as their paternal line (*93-11*) and maternal line (*PA64s*), respectively; these genes are classified as dominant expression.

### Functional analyses of differentially-expressed genes

**Figure 3:**
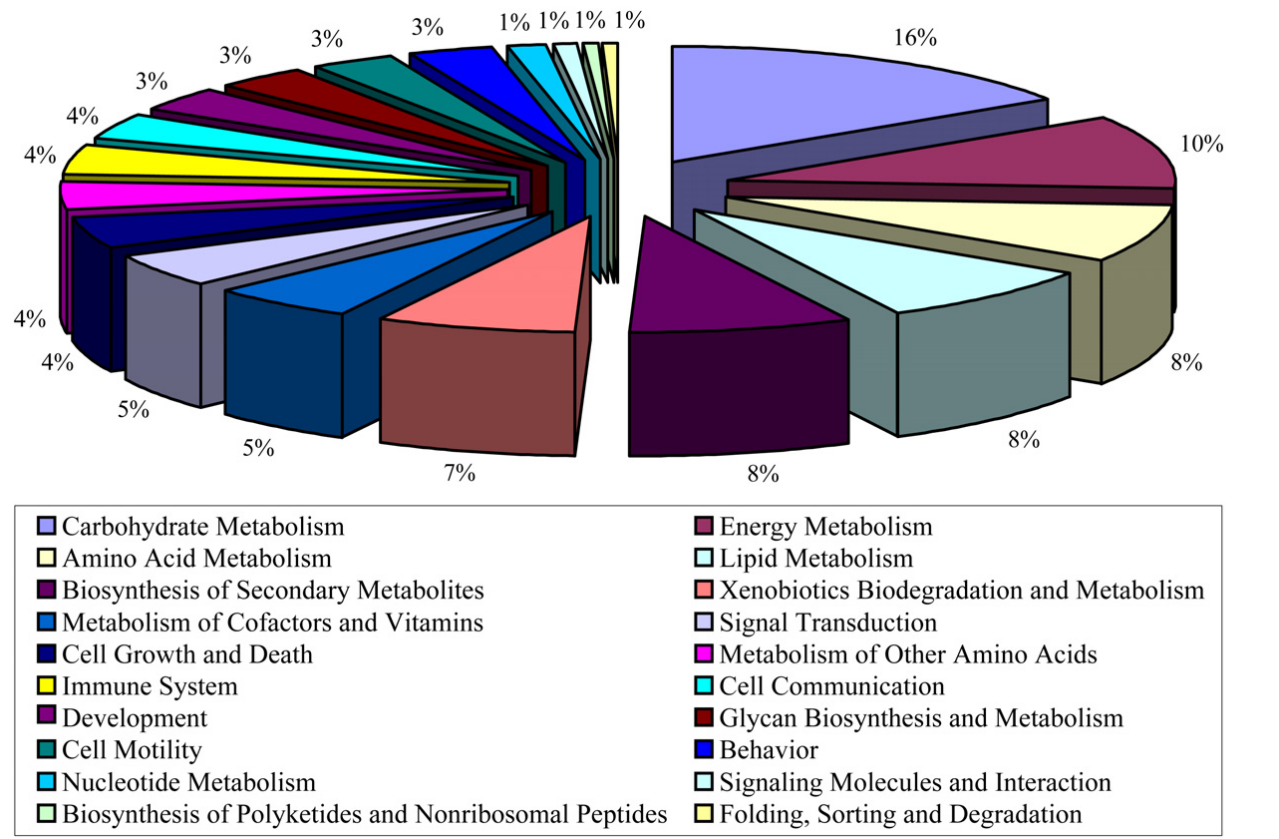
Functional categories of differentially-expressed genes (*P *< 0.05) among the three cultivars.

**Figure 4:**
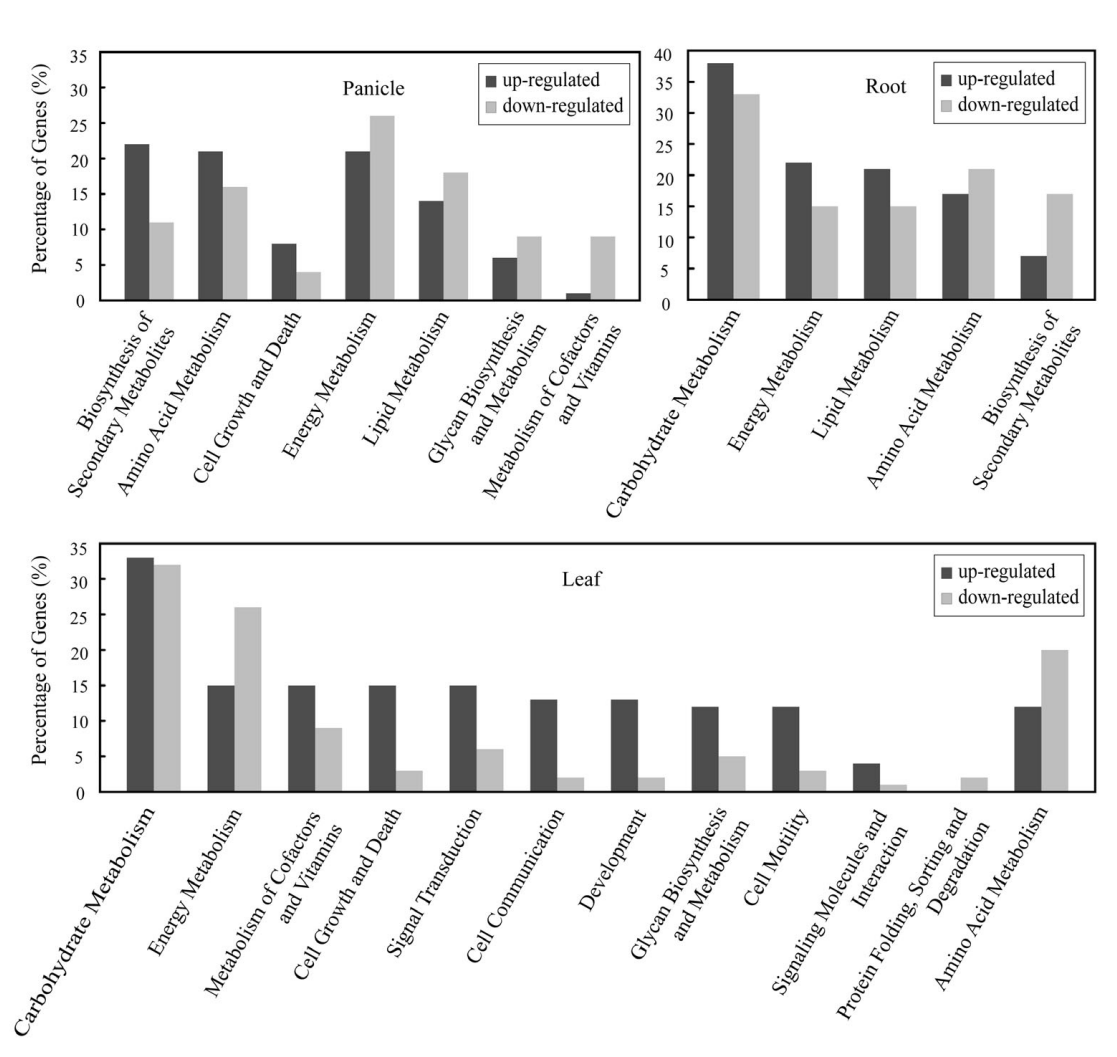
Functional Categories of up-regulated and down-regulated genes in panicles, leaves, and roots.

We annotated 217 (22.8%) and 850 (89.3%) differentially-expressed genes on the basis of two general databases, KEGG (Kyoto Encyclopedia of Genes and Genomes)[29] and InterPro/Network [30], respectively. The genes were further classified into 20 categories according to KEGG Gene Ontology (KOG) classification scheme (Figure 3); among them, genes involved in carbohydrate metabolism are the most abundant (16%), followed by energy metabolism (10%), and amino acid metabolism (8%). For instance, differentially-expressed genes in the hybrid are mostly related to enhancing carbon assimilation, energy metabolism, and biosynthesis of secondary metabolites; this effect is not due to simple distribution bias in the overall gene distribution since other categories were found decreased in the hybrid, such as protein sorting/folding/degradation in leaves (Figure 4). Dramatic down-regulation was also seen in metabolisms of co-factors and vitamins in panicles.

**Figure 5:**
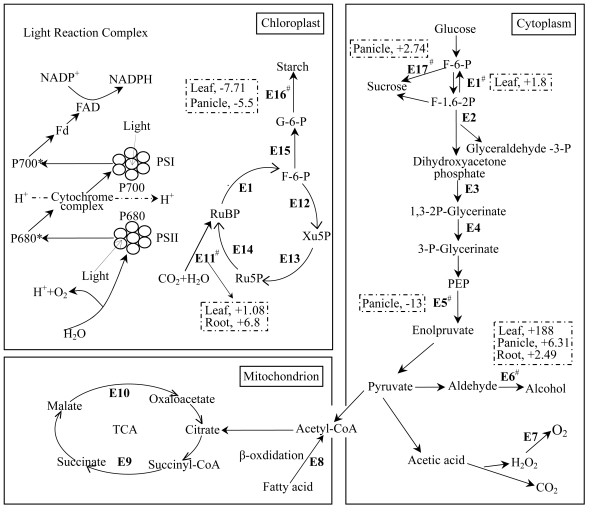
**Differentially-expressed genes that are involved in selected key metabolic pathways among three major cellular compartments**. Genes involved in photosynthesis, glycolysis/gluconeogenesis, citrate cycle (TCA cycle), anaerobic respiration, glycolic acid oxidation, and fatty acid β-oxdidation pathways are shown. The enzymes (^# ^denotes key or rate-limiting enzymes) are: E1^#^, fructose-1,6-bisphosphatase; E2, fructose-bisphosphate aldolase; E3, glyceraldehyde 3-phosphate dehydrogenase; E4, phosphoglycerate kinase; E5^#^, pyruvate kinase; E6^#^, alcohol dehydrogenase; E7, catalase; E8, acyl-CoA dehydrogenase; E9, succinyl-CoA ligase; E10, malate dehydrogenase; E11^#^, ribulose bisphosphate carboxylase; E12, transketolase; E13, ribulose-phosphate 3-epimerase; E14, phosphoribulokinase; E15, beta-phosphoglucomutase, 1,4-alpha-glucan branching enzyme; E16^#^, sucrose phosphate synthase; E17^#^, sucrose synthase. Proteins and enzymes in the light reaction complex are plastocyanin, ferredoxin [2Fe-2S], chlorophyll A-B binding protein, photosystem II protein PsbX, photosystem II protein PsbW, photosystem II protein PsbY, photosystem II oxygen evolving complex protein PsbP, photosystem II protein PsbR, photosystem II manganese-stabilizing protein PsbO, photosystem II oxygen evolving complex protein PsbQ, photosystem I reaction centre (subunit XI PsaL), photosystem I psaG/psaK protein, photosystem I reaction centre subunit N, photosystem I reaction center protein PsaF (subunit III), NADH:flavin oxidoreductase/NADH oxidase, and cytochrome b ubiquinol oxidase. The ratios of up- (+) or down (-) -regulated tags are indicated. Detailed information for light reaction complexes is listed in Additional file 6. Note that the key enzymes are either up- or down-regulated in three tissues; this behavior suggests active yet unique regulations in the hybrid.

Although the overall comparison to the previous results that were based on less number of tags led to similar conclusions, we feel that our current data allowed us to further look into more pathways and molecular details, which were not thoroughly exploited in the previous analysis. We divided carbon metabolism into three cellular compartments: the chloroplast, the mitochondrion, and the cytoplasm (Figure 5). The genes involved in photosynthesis in chloroplast were all up-regulated both in leaves and roots but down-regulated in panicles; this trend was readily observed in the overall distribution (Figure 2). Among them, 12 genes encode chlorophyll a/b binding proteins, 17 are photosystem I/II component genes, and ribulose bisphosphate carboxylase that is a key enzyme mediating the initial reaction of CO_2 _fixation. Details of genes involved in light reaction are listed (see Additional file 6). We also observed three key enzymes involved in five other selected key pathways (glycolysis/gluconeogenesis, citrate cycle, anaerobic respiration, glycolic acid oxidate, and fatty acid β-oxdidation) in the mitochondrion and cytoplasm. The first enzyme, alcohol dehydrogenates involved in the anaerobic respiration, is the most up-regulated gene in all three tissues. The second enzyme, fructose-1,6-bisphosphatase involved in gluconeogenesis, is up-regulated only in leaves. The last, pyruvate kinase that catalyzes phosphoenolpyruvate to form pyruvate and ATP (or decomposition of carbohydrate) is down-regulated both in leaves and panicles but not in roots. In addition, we observed that catalase, known to be involved in glycolic acid oxidate pathway (one of the three respiration pathways and unique to rice for better adapting its watery environment), is significantly up-regulated. Furthermore, along the pathway of synthesizing sucrose and its storage form (starch), we identified four genes, encoding beta-phosphoglucomutase, 1,4-alpha-glucan branching enzyme, sucrose phosphate synthase, and sucrose synthase, which are also up-regulated in leaves and panicles. These enzymes are believed to contribute to high grain yield in the super-hybrid rice.

There were many other functionally annotated genes found to be significantly up-regulated, including rapid alkalinization factor, proteinase inhibitor, and MADS-box transcription factors; all appeared to be relative to the traits for photoperiod sensitive genic male sterility, male fertility restoration, and pollen fertility, according to the quantitative trait loci (QTL) database (Gramene [31]; see Additional file 7). Among them, the MADS-box (9311_Chr06_3092 and 9311_Chr01_4641) and rapid alkalinization factor (9311_Chr12_1510) genes were found highly expressed in the hybrid as compared to its parental lines despite the fact that the expression of these genes are already higher in its paternal line *93-11 *than in its maternal line *PA64s*. This result indicated that these genes may play important roles directly or indirectly in flower morphogenesis and fertility of hybrid *LYP9*.

We also identified a large number of down-regulated genes that were not obvious in the previous analysis, largely due to more mapped tags and subtleties in data analysis protocols. These expression-suppressed genes belong to different functional categories among the three tissues; most of them are involved in energy metabolism, lipid metabolism, and glycan biosynthesis and metabolism in panicles, amino acid metabolism and protein processing in leaves, and biosynthesis of secondary metabolites in roots (Figure 4). The top-one down-regulated genes in panicles, leaves, and roots are metallothionein, peptidase M48, and glutathione S-transferase respectively. Metallothioneins are cysteine-rich proteins that can bind to heavy metals and scavenging reactive oxygen to protect plants from oxidative damage. Although it is the most down-regulated gene in panicle, it is up-regulated in root which plays an important role in assimilating, filtrating, and concentrating metal irons especially in screening heavy metal irons. Peptidase M48 is a family of proteins that function in protein degradation. We also found some other down-regulated genes related protein degradation, such as ubiquitin and ubiquitin-conjugating enzyme. Glutathione S-transferase is an enzyme to metabolize toxic exogenous compound that utilizes glutathione in the detoxification, for chemical defense in plants. We speculate that both of these up- and down-regulated genes represent a significant fraction of the genes regulating panicle development, rapid growth, stress tolerance, and grain yield in *LYP9*. Obviously, further verification and functional examination of these differentially-expressed genes are of essence in understanding their precise roles in heterosis.

### Cross-referencing SAGE data to Microarray-based results

We have compared our SAGE data with those from microarray-based experiments in a limited way where only data from one tissue, the leaf, were eligible for legitimate comparison, since the mRNA sample was harvested from leaves at the milking stage, identical to what we used for the SAGE experiment. The microarray data were acquired by using a custom-designed oligoarray that contains 60,727 oligonucleotide probes representing all predicted genes from the genome assembly of *93-11 *[22]. From this grand total, we identified 3,355 informative data points that were found in both microarray and SAGE data, and 2,312 (69%) of them showed a consistent trend between the two types of experiments (the spearman coefficient is 0.497, *P *< 0.0005). We found that the consistent trend among genes with a moderate-to-high expression between the two datasets correlated fairly well (the spearman coefficient is 0.743, P < 0.0005; data not shown). Of these genes, 222 (39%) were differentially-expressed according to the SAGE data with significance (*P *< 0.05). We listed 23 genes with a fold change of greater or equal to 2 in Table 5. These confirmation rates are not much different from reported comparative analyses between these two types of experiments since the reasons for systematic errors are multifold, including sampling time, experimental procedures, and data normalization [13].

**Table 5:** Differentially-expressed genes from *93-11 *leaf libraries confirmed by microarray data

Gene Model	Tag	Tag Number			Ratio^b^	Microarray Signal			Annotations
					
		N^a^	P^a^	L^a^		N^a^	P^a^	L^a^	
Up-Regulated Tags (≥2-fold)									
9311_Chr08_2156	GATTTGTATA	1	0	33	66.00	251	200	275	Plastocyanin-like
9311_Chr06_1523	TCATTTCAGT	2	0	14	14.00	3706	3473	6017	Major intrinsic protein
9311_Chr06_1142	ATCTGTTGCT	0	2	8	8.00	224	246	263	EPSP synthase (3-phosphoshikimate 1-carboxyvinyltransferase)
9311_Chr07_1712	GATCCGTCTC	13	0	47	7.23	1288	1238	2097	Thiamine biosynthesis Thi4 protein
9311_Chr06_1545	GTACTGTCTG	13	19	55	3.44	249	361	410	Ubiquitin
9311_Chr03_1401	TTCCCCCATT	11	4	22	2.93	261	150	263	Protein of unknown function DUF250
9311_Chr05_0842	CTGTATTACT	41	47	94	2.14	1030	994	1072	Calcium-binding EF-hand
Down-Regulated Tags (>2-fold)									
9311_Chr11_0807	GAATATTGGA	0	43	3	7.17	854	1030	976	Sucrose synthase
9311_Chr10_2185	TATCATTACA	40	169	19	5.50	2536	3225	1968	Mitochondrial substrate carrier
9311_Chr07_1231	CACATAAATT	38	26	6	5.33	3539	1750	957	Photosystem I reaction centre subunit IV/PsaE
9311_Chr03_0009	TACATAGACA	23	66	11	4.05	667	681	659	Unknown
9311_Chr03_3682	ATTGCGGAAT	103	323	55	3.87	4577	5270	3054	Glycine hydroxymethyl transferase
9311_Chr01_4972	GATCGATGGG	4	53	8	3.56	239	747	504	Cellular retinaldehyde-binding)/triple function, C-terminal
9311_Chr03_3625	ACACTACAGT	2	36	6	3.17	203	401	245	Unknown
9311_Chr03_4144	CTTACAAGTG	25	58	14	2.96	929	947	655	Rieske [2Fe-2S] region
9311_Chr01_2088	GAGAGAGGGA	117	186	52	2.91	6807	7259	3098	Photosystem II manganese-stabilizing protein PsbO
9311_Chr12_1000	GATATATGGA	69	256	58	2.80	2501	2801	1201	Photosystem I reaction centre, subunit XI PsaL
9311_Chr04_3185	TAGTGATAAG	8	36	8	2.75	1563	1689	1217	Lipase, class 3
9311_Chr03_0940	ATCGCCGAGA	19	68	17	2.56	1520	2064	1220	Glutamine synthetase, beta-Grasp
9311_Chr01_4844	GTTAGCAAAA	11	17	6	2.33	2280	2985	1878	Calsequestrin
9311_Chr06_2649	AGGGAGGCCG	25	2	6	2.25	246	192	222	Heat shock protein DnaJ, N-terminal

^a ^P, N, and L stand for *PA64s*, *9311*, and *LYP9*, respectively. ^b ^Ratios are calculated as ratio = L/[(P+N)/2] for up-regulated tags and [(P+N)/2]/L for down-regulated tags.

## Discussion

### Tag-to-gene mapping procedures

SAGE and related sequencing-based techniques are very effective for studying gene expression in organisms where well-characterized genome sequences are available, and they have been applied to a number of eukaryotic species [17,19,32] and the merits and success have been discussed very recently by Marco Marra and his colleagues with ample experimental data [12], albeit pitfalls do exist [13]. In our previous SAGE study, we utilized the available FL-cDNA sequences [24] for tag-to-gene mapping [21], as these FL-cDNA sequences best represent the rice transcriptome albeit in a rather limited amount. However, a large proportion (83%) of the SAGE tags was not found in this cDNA data collection that is known not covering all the genes of the rice genome. To overcome this limit, we utilized a new strategy for tag-to-gene mapping based on newly annotated genes of the two rice genome assemblies and other transcript sequences (FL-cDNA, UniGene, and ESTs). This process led to a significant improvement in gene identification, resulting in 10,268 additional tags and 68.85% extra differentially-expressed genes at a higher *P *value (*P *< 0.01), as compared to the previous collection.

Aside from the success of mapping SAGE tags to annotated genes in the genome, there are a couple of important points that are worthy of further discussion. First, we always have tags that are mapped to ambiguous positions, and they may belong to multiple loci (such as gene families and splicing variants) in the genome sequence, especially when the length of SAGE tags is as short as 14 bp. There were 4,014 (20%) such tags in our case, we assigned these tags to the genomes and used them for functional analysis. For example, despite the fact that a tag with a sequence of "AACAAGCTCA" was assigned to two different loci (9311_Chr04_1718 and 9311_Chr05_1829), the two were evidenced by two different FL-cDNA sequences (AK0ah71547 and AK061050), allowing us to identify them as members of the fructose-bisphosphate aldolase gene family. These two genes were found down-regulated in roots of the hybrid line, and they are involved in glycolysis/Gluconeogenesis pathways. Therefore, it is critical to map these seemingly ambiguous genes, especially when they are differentially regulated in the hybrid. It is possible to design experiments to distinguish these genes with locus-specific primers since most of these duplicated (or closely related) genes may be not identical in their UTR and genomic sequence, especially when genome sequences are readily available. As we have reported previously, the rice genome has enormous number of duplicated genes [23] that some of them may actually hold pivotal information in hybrid vigor.

The second point has to do with the fact that a fraction (often more than 40%) of the experimental tags remains unassigned to genes so we need to figure out the possible reasons. When comparing unassigned tags to virtual tags based on predicted *Nla*III sites in the nuclear and organellar (mitochondrial and chloroplast) genome sequences, we found that 2,500 tags out of 47,867 (5%) were absent in the genome sequence assembly of *93-11*, and 342 tags (0.6%) were derived from either the mitochondrial (491 kb) or chloroplast genomes (134 kb). These unassigned tags are most likely due to sequencing errors, sequences interrupted by introns, un-assembled sequences (including those in the sequence gaps), and organelle-specific sequences. In addition, we have technically implemented an artificial 300-bp UTRs for predicted genes without transcript-based evidence and only extracted the 3' most (canonical position) tags from virtual transcripts. This procedure is certainly incapable of including all UTR length variants, largely due to the absence of canonical polyadenylation signal for the accurate determination of the 3' UTR length in plant genomes [33]. To estimate the result of such a procedure, we compared the remaining total unassigned tags to a cumulative virtual tag dataset constructed by varying the artificial UTR lengths in a 100-bp interval, from 100 to 500 bp, resulting in a further assignment of 3,119 (6.5%) additional tags. However, these tags were considered unreliable and were not included in this analysis. Nevertheless, the UTR-derived anomaly seems contributing to the impaired tag assignment in a similar way as the sequence anomaly. Other obvious factors resulting in unassigned tags, such as experimental artifacts (incomplete enzyme digestions and ligations, as well as inefficient cloning procedures), are not discussed here in details.

### The differentially-expressed genes in multiple expression patterns

Over the years, differential gene expression between the hybrid and its parental cultivars has been hypothesized to attribute to heterosis [5,34]. As having partitioned the differentially-expressed genes into twelve patterns as conventionally done, we found only 25% to 45% or minorities of the genes were additively expressed in the rice hybrid; this result contradicted what was reported for a similar study in hybrid maize, where additively expressed genes were found as a major trend, 77.7% [35]. The reason for such a disparity may be complex as it may be related to operational pollination strategies and differences in epigenetic regulations. Meyer et al. (2004) have shown that alternative pollination methods (hand-vs. self-pollination) have significant effects on seed size and early seedling growth rate in Arabidopsis. The patterns of gene expression altered obviously in cross-fertilized kernel as compared to self-fertilized kernel, both qualitatively and quantitatively [36], largely due to cis-transcriptional variations in maize inbred lines that lead to additive expression patterns in the F1 hybrids [37]. For the involvement of possible epigenetic mechanisms, we refer to the difference in transposon density between the two species as the maize genome is more heavily bombarded by active repeats and we speculate that a more vigorous methylation tactic might be used in gene regulation in maize. Among non-additively expressed genes, both over-dominant and under-dominant genes are rather abundant, supporting in part the over-dominance hypothesis for rice heterosis [34].

Among all differentially-expressed genes, we identified up to 70% of them (*P *< 0.01) exhibiting paternal-like expression (PLE) profiles, especially in panicles, which are at least in part attributable to two plausible mechanisms – molecular imprinting and defective expressions of the maternal alleles – as often observed in panicles harvested at the pollen maturing stage, where thermo-sensitive male sterility of the maternal line (*PA64s*) may be relevant [38]. For instance, two MADS-box transcription factors related to pollen fertility have been consistently observed as up-regulated in the hybrid, but they do not express in the male-sterility plant [39,40]. The rapid alkalinization factor, a polypeptide hormone that was suggested to be related to nuclear sterility and development [41], was observed to be up-regulated and located in photoperiod-sensitive and genic male sterility trait based on our QTL analysis. Although we have not been able to plot plausible functional scenarios on the precise roles of these genes, the findings undoubtedly provide useful clues for future molecular studies.

### Putative regulation mechanisms of differentially-expressed genes

Differential gene expression in plants is known to be mainly regulated by two forms of mechanisms – cis- and trans-regulations at transcription levels as well as epigenetic and post-transcription modulations [6]. For instance, differential methylation in CpG or CNG islands [9,42] and allele-dependent mechanisms of gene regulation [43] have been demonstrated between hybrid and its parents in rice and maize. However, variations among cis-regulatory elements are hard to study but trans-regulatory factors are easier to identify based on gene expression data. We have indeed found over 48 transcription factors, annotated as differentially-expressed genes, including MADS-box genes, TFIIE, bZIP, and Jumonji; these genes have been found involved in various aspects of development and differentiation in land plants. Some of the MADS-box genes function in floral tissues as "molecular architects" of flower morphogenesis. TFIIE is an essential component of the RNA polymerase II transcription machinery [44], playing important roles at two distinct but sequential steps in transcription: pre-initiation complex formation-activation (open complex formation) and the transition from initiation to elongation [45]. Although the possible contributions of these transcription factors, all-purpose or members of multiple gene families, to hybrid vigor may not be easily demonstrated, their presence and regulated expression are initial clues for in-depth molecular and genetic studies.

An increasing number of studies have reported that functional divergence in duplicated gene is accompanied by gene expression change although the evolution mechanism behind this process remains unclear. There was a report that 7% of duplicated gene pairs co-express in yeast [46], and we know that gene and chromosomal segment duplications widely exist in the rice genome, including an ancient whole genome duplication, recent segmental duplications, and massive ongoing individual gene duplications that cover 65.7% of the genome [23]. We found 7 of our 698 ambiguous assigned tags are mapped to the duplicated gene pairs, which we suspected the duplication with a high homology may affect gene expression including silencing and up- or down-regulation of one of the duplicated genes after hybridization [47]. When looking into the possible molecular assays in distinguishing the different alleles, we found that it is actually possible to design allele-specific primers to detect the expression level of duplication pairs.

## Conclusion

We improved the tag-to-gene mapping strategy by combining information from transcript sequences and rice genome annotation and obtained over 10,000 new tags for a more comprehensive view of genes that related to rice heterosis. These heterotic expression genes among different genotypes provided new avenues for exploring the molecular mechanisms underlying heterosis, including variable gene expression patterns.

## Methods

### PCUE database

We constructed a PCUE database for rice (*Oryza sativa*) on the basis of available genomic resources that contain (1) the improved whole genome shot-gun sequence assemblies of *93-11 *[GenBank: AAAA02000000] and *PA64s *as well as their annotations [48], (2) a collection of 19,079 non-redundant FL-cDNAs (nr-FL-cDNAs; [23] from KOME [49], and (3) 51,336 UniGenes (UniGene Build #59) and 1,183,931 ESTs from NCBI [50].

We aligned the collected transcript sequences to the two genome sequences by using BLAT [51] to obtain a dataset for tag annotations. The threshold parameters set for aligned transcripts are (1) at least 90% identical to their genomic sequences and (2) covering ≥ 90% transcript sequences. When a transcript has more than one hit to genomic sequences, the longest consensus was selected as the best-aligned (true) locus. We further selected sequences that span the 3' end of a predicted gene but do not extend to the next with ≥ 100-bp overlapping sequences. As a result, our predicted genes were partitioned into two sets: supported by one or more transcripts and without supporting data.

### The evaluation dataset

In order to evaluate the accuracy of tag-to-gene mapping methodology, we built a test dataset that contains 2,480 FL-cDNA sequences that satisfied all five criteria: (1) ORF length > 300 bp, (2) with poly(A) signal (AATAAA/ATTAAA) or poly(A) tails (with a minimal number of five A) [15], (3) alignable to a unique predicated gene with homolog (based on 50% protein sequence similarity or 100 residues) to Arabidopsis, (4) a unique CATG tag and experimental data, and (5) alignable to a unique predicted gene and corresponding UniGenes or ESTs. We further divided this dataset into three categories: UniGene, EST, and predicted gene. In the Unigene and EST categories, we have twelve subsets. Eight of those were sequences with poly(A) signal (Uni-S and EST-S), with poly(A) tails (Uni-A and EST-A), with both poly(A) signal and tail (Uni-B and EST-B), without poly(A) signal and tails (Uni-N and EST-N). The other four subsets contained the longest and the best transcripts that were best validated by either UniGenes or ESTs (Unibest or ESTbest). To know the length of 3'-UTR, we used 19,079 non-redundant FL-cDNA to determine the length distribution and found that 95% of these genes have UTR length shorter than 1280 bp, with an average size of 422 bp and a median of 295 bp. We therefore added five different lengths (100-, 200-, 300-, 400-, and 500-bp) to construct virtual UTRs for the predicted genes. We finally built virtual tags from each of the above-mentioned subsets by extracting a 10-bp tag from the immediate downstream sequence of the last (3'-most) *Nla*III (CATG) site. We evaluated the success rates of virtual tags that match the test set.

### Virtual tags and tag-to-gene mapping

Since predicted genes do not have UTRs, we extracted consecutive exons together to form gene models from the two genome assemblies and added to them either UTR sequences based on information from known transcripts or artificial UTRs in a length of 300 bp. We obtained four groups of tag data, including those based on cDNA, Unimax, ESTmax, and predicted genes (P-300). We mapped 68,462 unique empirical tags from our data [21] to the four groups of virtual tags after filtering cloning linkers, vectors, and simple repeats. We excluded 47,867 tags from further processing and their outcomes from our analysis protocol were summarized (see Additional file 8). These tags were regarded as unmapped tags although 45,025 of them were actually mapped to the nuclear genome but in unexpected range of correct positions of exon and UTR sequences. Most of them were believed to fragmented mRNAs that were co-processed during library construction procedures.

We annotated all our SAGE tags based on InterPro/Network and KEGG for protein families, domains, and functions. We chose the best scoring primary (sequence similarity-based) annotations from family-type categories first, followed by domain-type and others. If the gene had no primary annotation then we used a network-based annotation [52]. *P *values between copy numbers among libraries were calculated based on Audic-Claverie (or AC) statistics [28] by using IDEG6 software [53,54]. The significance of the differentially-expressed genes was defined with *P *values less than 0.05 or 0.01. Ratios of up-regulated and down-regulated genes were calculated according to ratio = L/[(P+N)/2] (≥ 2) and [(P+N)/2]/L (<2), respectively.

### Microarray and QTL data

We used microarray data from the leaf tissue at the milky stage, which were generated in our laboratory. The microarray contains 60,727 oligonucleotide probes representing all predicted genes from the genome sequence of *93-11 *[22]. We physically mapped the oligonucleotides to the most up-to-date version of the genome assembly [48] with the threshold that each oligonucleotide must match to one unique gene with 90% or higher sequence identity. We also used rice QTL data with physical position on TIGR4 genome from Gramene [31]and mapped differentially-expressed genes to nine QTL categories.

## Abbreviations

PLE, Paternal-like expression; MLE, Maternal-like expression; SAGE, Serial analysis of gene expression; QTL, Quantitative trait locus; nr-FL-cDNAs, non-redundant full-length cDNAs

## Competing interests

The author(s) declares that there are no competing interests.

## Authors' contributions

SHS and HZQ conceived and carried out the study design, performed the bioinformatics analysis, interpreted the analysis results and drafted the manuscript. CC, SNH, and JY participated in the study design and helped in revising and editing the manuscript. All authors read and approved the final manuscript.

## Supplementary Material

Additional file 1 Size distributions of UTR based on known FL-cDNAs for 5'-UTRs (A) and 3'-UTRs (B). Using the known full-length cDNA sequences from KOME database, we plotted the size distribution of UTRs to determine the artificial UTR length. Click here for file

Additional file 2 The details of differentially-expressed genes in panicle. We integrated expression information, function annotation, and tag-mapping information of panicle differentially-expressed genes in the excel tables. Click here for file

Additional file 3 The details of differentially-expressed genes in leaf. We integrated expression information, function annotation, and tag-mapping information of leaf differentially-expressed genes in the excel tables. Click here for file

Additional file 4 The details of differentially-expressed genes in root. We integrated expression information, function annotation, and tag-mapping information of root differentially-expressed genes in the excel tables. Click here for file

Additional file 5 Differentially-expressed tags (P < 0.05) that exhibit changes of >16 folds and that were mapped to PA64s only but not plotted in Figure 3. Lists of genes that do not reflected in Figure 2. Click here for file

Additional file 6 Genes involved in the light reaction. We categorized and listed the genes involved in the light reaction. Click here for file

Additional file 7 Sterility and fertility-related differentially-expressed genes in panicle. By comparing to the Gramene QTL database, the Sterility and fertility-related differentially-expressed genes in panicle were listed. Click here for file

Additional file 8 The statistic result of the potential origin of SAGE tags that were not mapped in this analysis. An example of the potential origin of SAGE tags that were not mapped in this analysis was got by directly mapping tags to genome sequences. Click here for file

## References

[B1] Budak H (2002). Understanding of Heterosis. KSU J Science and Engineering.

[B2] Xiao J, Li J, Yuan L, Tanksley SD (1995). Dominance is the major genetic basis of heterosis in rice as revealed by QTL analysis using molecular markers. Genetics.

[B3] Yu SB, Li JX, Xu GC, Tan YF, Gao YJ, Li XH, Zhang QF, Maroof MAS (1997). Importance of epistasis as the genetic basis of heterosis in an elite rice hybrid. Proc Natl Acad Sci USA.

[B4] Birchler JA, Auger DL, Riddle NC (2003). In search of the molecular basis of heterosis. Plant Cell.

[B5] Sun Q, Wu L, Ni Z, Meng F, Wang Z, Lin Z (2004). Differential gene expression patterns in leaves between hybrids and their parental inbreds are correlated with heterosis in a wheat diallel cross. Plant Science (Oxford).

[B6] Yao Y, Ni Z, Zhang Y, Chen Y, Ding Y, Han Z, Liu Z, Sun Q (2005). Identification of differentially expressed genes in leaf and root between wheat hybrid and its parental inbreds using PCR-based cDNA subtraction. Plant Mol Biol.

[B7] Wu LM, Ni ZF, Meng FR, Lin Z, Sun QX (2003). Cloning and characterization of leaf cDNAs that are differentially expressed between wheat hybrids and their parents. MGG Molecular Genetics and Genomics.

[B8] Yao Y, Ni Z, Du J, Wang X, Wu H, Sun Q (2006). Isolation and characterization of 15 genes encoding ribosomal proteins in wheat (Triticum aestivum L.). Plant Science (Oxford).

[B9] Xiong LZ, Xu CG, Saghai Maroof MA, Zhang Q (1999). Patterns of cytosine methylation in an elite rice hybrid and its parental lines, detected by a methylation-sensitive amplification polymorphism technique. Mol Gen Genet.

[B10] Ni Z, Sun Q, Liu Z, Wu L, Wang X (2000). Identification of a hybrid-specific expressed gene encoding novel RNA-binding protein in wheat seedling leaves using differential display of mRNA. Mol Gen Genet.

[B11] Huang Y, Zhang L, Zhang J, Yuan D, Xu C, Li X, Zhou D, Wang S, Zhang Q (2006). Heterosis and polymorphisms of gene expression in an elite rice hybrid as revealed by a microarray analysis of 9198 unique ESTs. Plant Mol Biol.

[B12] Khattra J, Delaney AD, Zhao Y, Siddiqui A, Asano J, McDonald H, Pandoh P, Dhalla N, Prabhu AL, Ma K, Lee S, Ally A, Tam A, Sa D, Rogers S, Charest D, Stott J, Zuyderduyn S, Varhol R, Eaves C, Jones S, Holt R, Hirst M, Hoodless PA, Marra MA (2007). Large-scale production of SAGE libraries from microdissected tissues, flow-sorted cells, and cell lines. Genome Res.

[B13] Wang SM (2007). Understanding SAGE data. Trends Genet.

[B14] Chen J, Sun M, Lee S, Zhou G, Rowley JD, Wang SM (2002). Identifying novel transcripts and novel genes in the human genome by using novel SAGE tags. Proc Natl Acad Sci USA.

[B15] Boon K, Osorio EC, Greenhut SF, Schaefer CF, Shoemaker J, Polyak K, Morin PJ, Buetow KH, Strausberg RL, De Souza SJ, Riggins GJ (2002). An anatomy of normal and malignant gene expression. Proc Natl Acad Sci USA.

[B16] Lee JY, Lee DH (2003). Use of serial analysis of gene expression technology to reveal changes in gene expression in Arabidopsis pollen undergoing cold stress. Plant Physiol.

[B17] Fizames C, Munos S, Cazettes C, Nacry P, Boucherez J, Gaymard F, Piquemal D, Delorme V, Commes T, Doumas P, Cooke R, Marti J, Sentenac H, Gojon A (2004). The Arabidopsis root transcriptome by serial analysis of gene expression. Gene identification using the genome sequence. Plant Physiol.

[B18] Matsumura H, Nirasawa S, Terauchi R (1999). Technical advance: transcript profiling in rice (Oryza sativa L.) seedlings using serial analysis of gene expression (SAGE). Plant J.

[B19] Gibbings JG, Cook BP, Oufault MR, Madden SL, Khurie S, Tumbull CJ, Dunwell JM (2003). Globle transcript analysis of rice leaf and seed using SAGE technology. Plant Biotechnology Journal.

[B20] Wang Q, Zhang QD, Jiang GM, Lu CM, Kuang TY, Wu S, Li CQ, Jiao DM (2000). Photosynthetic Characteristics of Two Superhigh-yield Hybrid Rice. Acta Botanica Sinica.

[B21] Bao J, Lee S, Chen C, Zhang X, Zhang Y, Liu S, Clark T, Wang J, Cao M, Yang H, Wang SM, Yu J (2005). Serial analysis of gene expression study of a hybrid rice strain (LYP9) and its parental cultivars. Plant Physiol.

[B22] Yu J, Hu S, Wang J, Wong GK, Li S, Liu B, Deng Y, Dai L, Zhou Y, Zhang X, Cao M, Liu J, Sun J, Tang J, Chen Y, Huang X, Lin W, Ye C, Tong W, Cong L, Geng J, Han Y, Li L, Li W, Hu G, Huang X, Li W, Li J, Liu Z, Li L (2002). A draft sequence of the rice genome (Oryza sativa L. ssp. indica). Science.

[B23] Yu J, Wang J, Lin W, Li S, Li H, Zhou J, Ni P, Dong W, Hu S, Zeng C, Zhang J, Zhang Y, Li R, Xu Z, Li S, Li X, Zheng H, Cong L, Lin L, Yin J, Geng J, Li G, Shi J, Liu J, Lv H, Li J, Wang J, Deng Y, Ran L, Shi X (2005). The Genomes of Oryza sativa: a history of duplications. PLoS Biol.

[B24] Kikuchi S, Satoh K, Nagata T, Kawagashira N, Doi K, Kishimoto N, Yazaki J, Ishikawa M, Yamada H, Ooka H, Hotta I, Kojima K, Namiki T, Ohneda E, Yahagi W, Suzuki K, Li CJ, Ohtsuki K, Shishiki T, Otomo Y, Murakami K, Iida Y, Sugano S, Fujimura T, Suzuki Y, Tsunoda Y, Kurosaki T, Kodama T, Masuda H, Kobayashi M (2003). Collection, mapping, and annotation of over 28,000 cDNA clones from japonica rice. Science.

[B25] Wu J, Maehara T, Shimokawa T, Yamamoto S, Harada C, Takazaki Y, Ono N, Mukai Y, Koike K, Yazaki J, Fujii F, Shomura A, Ando T, Kono I, Waki K, Yamamoto K, Yano M, Matsumoto T, Sasaki T (2002). A comprehensive rice transcript map containing 6591 expressed sequence tag sites. Plant Cell.

[B26] Tang J, Xia H, Li D, Cao M, Tao Y, Tong W, Zhang X, Hu S, Wang J, Yu J, Yang H, Zhu L (2005). Gene expression profiling in rice young panicle and vegetative organs and identification of panicle-specific genes through known gene functions. Mol Genet Genomics.

[B27] Gene Expression Omnibus. http://www.ncbi.nlm.nih.gov/geo/.

[B28] Audic S, Claverie JM (1997). The significance of digital gene expression profiles. Genome Res.

[B29] Kyoto Encyclopedia of Genes and Genomes. http://www.genome.jp/kegg/.

[B30] InterPro. http://www.ebi.ac.uk/interpro/.

[B31] Gramene. http://www.gramene.org.

[B32] Pleasance ED, Marra MA, Jones SJ (2003). Assessment of SAGE in transcript identification. Genome Res.

[B33] Graber JH, Cantor CR, Mohr SC, Smith TF (1999). In silico detection of control signals: mRNA 3'-end-processing sequences in diverse species. Proc Natl Acad Sci USA.

[B34] Tsaftaris SA (1995). Molecular aspects of heterosis in plants. Plant Physiol.

[B35] Swanson-Wagner RA, Jia Y, DeCook R, Borsuk LA, Nettleton D, Schnable PS (2006). All possible modes of gene action are observed in a global comparison of gene expression in a maize F1 hybrid and its inbred parents. Proc Natl Acad Sci USA.

[B36] Meng F, Ni Z, Wu L, Sun Q (2005). Differential gene expression between cross-fertilized and self-fertilized kernels during the early stages of seed development in maize. Plant Science.

[B37] Stupar RM, Springer NM (2006). Cis-transcriptional variation in maize inbred lines B73 and Mo17 leads to additive expression patterns in the F1 hybrid. Genetics.

[B38] Song LQ, Fu TD, Tu JX, Ma CZ, Yang GS (2006). Molecular validation of multiple allele inheritance for dominant genic male sterility gene in Brassica napus L. Theor Appl Genet.

[B39] Nagasawa N, Miyoshi M, Sano Y, Satoh H, Hirano H, Sakai H, Nagato Y (2003). SUPERWOMAN1 and DROOPING LEAF genes control floral organ identity in rice. Development.

[B40] Kang HG, Jeon JS, Lee S, An G (1998). Identification of class B and class C floral organ identity genes from rice plants. Plant Mol Biol.

[B41] Haruta M, Constabel CP (2003). Rapid alkalinization factors in poplar cell cultures. Peptide isolation, cDNA cloning, and differential expression in leaves and methyl jasmonate-treated cells. Plant Physiol.

[B42] Dai Y, Ni Z, Dai J, Zhao T, Sun Q (2005). Isolation and expression analysis of genes encoding DNA methyltransferase in wheat (Triticum aestivum L.). Biochimica et Biophysica Acta.

[B43] Hollick JB, Patterson GI, Asmundsson IM, Chandler VL (2000). Paramutation alters regulatory control of the maize pl locus. Genetics.

[B44] Maxon ME, Goodrich JA, Tjian R (1994). Transcription factor IIE binds preferentially to RNA polymerase IIa and recruits TFIIH: a model for promoter clearance. Genes Dev.

[B45] Okamoto T, Yamamoto S, Watanabe Y, Ohta T, Hanaoka F, Roeder RG, Ohkuma Y (1998). Analysis of the role of TFIIE in transcriptional regulation through structure-function studies of the TFIIEbeta subunit. J Biol Chem.

[B46] Zhang Z, Gu J, Gu X (2004). How much expression divergence after yeast gene duplication could be explained by regulatory motif evolution?. Trends Genet.

[B47] Adams KL, Wendel JF (2005). Novel patterns of gene expression in polyploid plants. Trends Genet.

[B48] Rice Information System. http://rise.genomics.org.cn/rice/index2.jsp.

[B49] Knowledge-based Oryza Molecular biological Encyclopedia. http://cdna01.dna.affrc.go.jp/cDNA/.

[B50] National Center for Biotechnology Information. http://www.ncbi.nih.gov/.

[B51] Kent WJ (2002). BLAT – the BLAST-like alignment tool. Genome Res.

[B52] McDermott J, Bumgarner R, Samudrala R (2005). Functional annotation from predicted protein interaction networks. Bioinformatics.

[B53] IDEG6. http://telethon.bio.unipd.it/bioinfo/IDEG6/.

[B54] Romualdi C, Bortoluzzi S, Danieli GA (2001). Detecting differentially expressed genes in multiple tag sampling experiments: comparative evaluation of statistical tests. Hum Mol Genet.

