# Supplementary figures and images for: Differential gene expression in an elite hybrid rice cultivar (Oryza sativa, L) and its parental lines based on SAGE data
Source: BMC Plant Biol. 2007 Sep 19;7:49. doi: 10.1186/1471-2229-7-49 (PMC2077334; doi:10.1186/1471-2229-7-49)

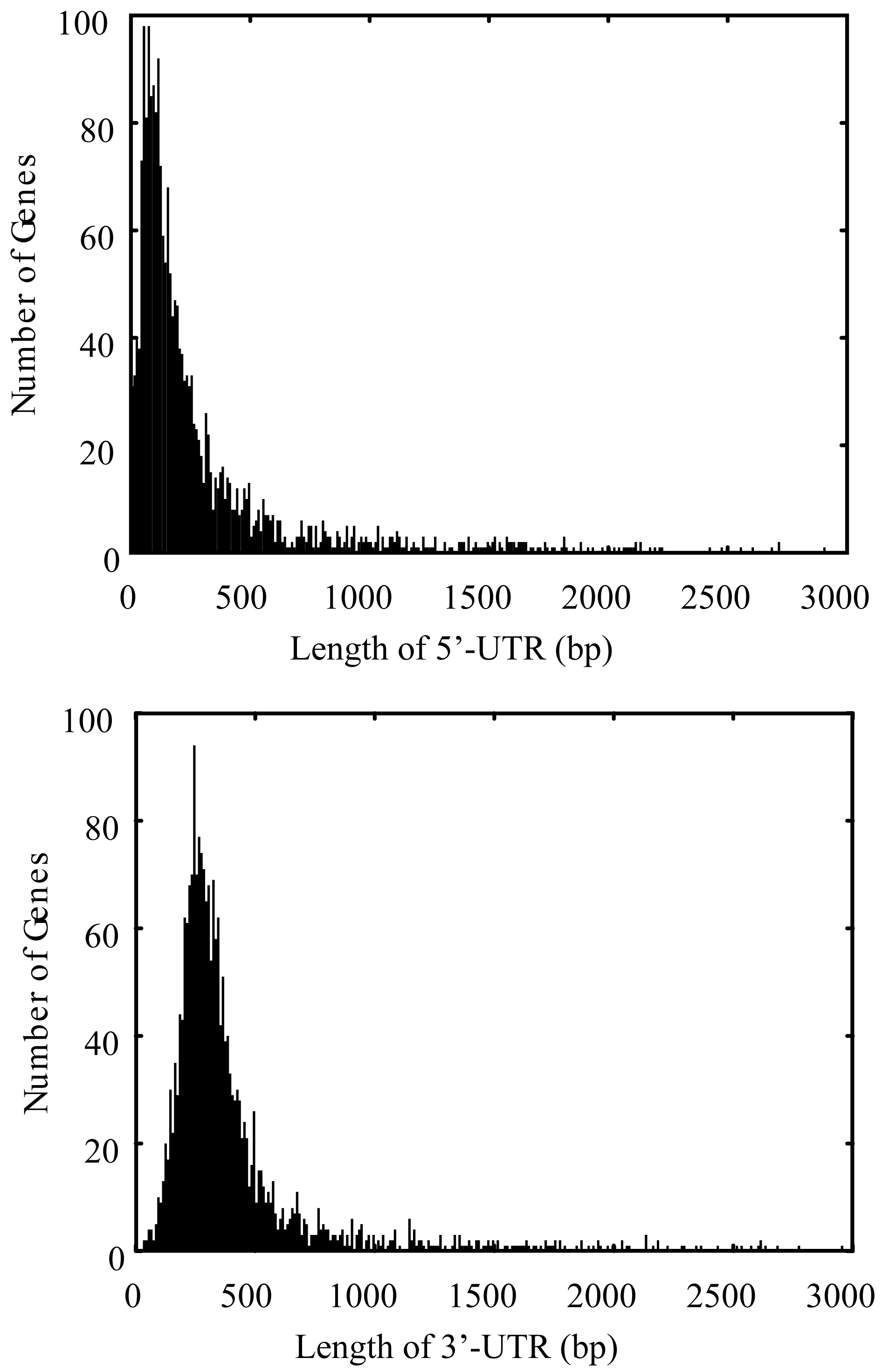

Supplement: Additional file 1 — Size distributions of UTR based on known FL-cDNAs for 5'-UTRs (A) and 3'-UTRs (B). Using the known full-length cDNA sequences from KOME database, we plotted the size distribution of UTRs to determine the artificial UTR length. [file 1471-2229-7-49-S1.tiff]
